# Titanium versus absorbable tacks comparative study (TACS): a multicenter, non-inferiority prospective evaluation during laparoscopic repair of ventral and incisional hernia: study protocol for randomized controlled trial

**DOI:** 10.1186/s13063-015-0779-x

**Published:** 2015-06-04

**Authors:** Gianfranco Silecchia, Giuseppe Cavallaro, Luigi Raparelli, Stefano Olmi, Gianandrea Baldazzi, Fabio Cesare Campanile

**Affiliations:** Department of Medico-Surgical Sciences and Biotechnologies, Sapienza University of Rome, Rome, Italy; General Surgery Unit, ICOT Hospital, Latina, LT Italy; General Surgery Unit, GB Grassi Hospital, Ostia, RM Italy; General Surgery Unit, Gruppo Ospedaliero San Donato, Milan, Italy; General and Mini-Invasive Surgery Unit, Abano Terme Hospital, Abano Terme, PD Italy; General Surgery Unit, Andosilla Hospital, Civita Castellana, VT Italy

**Keywords:** Ventral/incisional hernia repair, Laparoscopy, Lightweight polypropylene mesh, Absorbable tacks, Titanium helicoidal tacks, Hernia

## Abstract

**Background:**

Laparoscopic repair of ventral and incisional hernias has gained popularity since many studies have reported encouraging results in terms of outcomee and recurrence. Choice of mesh and fixation methods are considered crucial issues in preventing recurrences and complications. Lightweight meshes are considered the first choice due to their biomechanical properties and the ability to integrate into the abdominal wall. Titanium helicoidal tacks still represent the “gold standard” for mesh fixation, even if they have been suggested to be involved in the genesis of post-operative pain and complications. Recently, absorbable tacks have been introduced, under the hypothesis that there will be no need to maintain a permanent fixation device after mesh integration. Nevertheless, there is no evidence that absorbable tacks may guarantee the same results as titanium tacks in terms of strength of fixation and recurrence rates. The primary end point of the present trial is to test the hypothesis that absorbable tacks are non-inferior to titanium tacks in laparoscopic incisional and ventral hernia repair (LIVHR) by lightweight polypropylene mesh, in terms of recurrence rates at 3-year follow-up. Surgical complications, post-operative stay, comfort and pain are secondary end points to be assessed.

**Methods/Design:**

Two hundred and twenty patients with ventral hernia will be randomized into 2 groups:

Group A (110) patients will be submitted to LIVHR by lightweight polypropylene mesh fixed by titanium tacks;

Group B (110) patients will be submitted to LIVHR by lightweight polypropylene mesh fixed by absorbable tacks.

**Discussion:**

A few retrospective studies have reported similar results when comparing absorbable versus non-absorbable tacks in terms of intraoperative and early post-operative outcomes. These studies have the pitfalls to be retrospective evaluation of small series of patients, and the reported results still need to be validated by larger series and prospective studies.

The aim of the present trial is to investigate and test the non-inferiority of absorbable versus non-absorbable tacks in terms of hernia recurrence rates, in order to assess whether the use of absorbable tacks may achieve the same results as non-absorbable tacks in mid-term and long-term settings.

**Trial registration number:**

NCT02076984: 5 June 2014 (ClinicalTrials.gov)

**Electronic supplementary material:**

The online version of this article (doi:10.1186/s13063-015-0779-x) contains supplementary material, which is available to authorized users.

## Background

During the past 50 years, incisional and ventral hernia repair surgery has evolved from direct suture repair to the use of synthetic mesh to obtain a tension-free repair [1–3]. Finally, the tension-free concepts have been applied to laparoscopic surgery. Laparoscopic repair of incisional/ventral hernias (LIVHR) has gained increasing popularity, since many studies, included three meta-analyses, have reported encouraging results in terms of wound infection, hospital stay and post-operative pain, and recurrence rates comparable with the open approach [1–8].

Besides patient selection (age, sex, comorbidities, obesity, hernia site and size, eventual recurrence and type of previous abdominal surgery) and patient compliance, the choice of the mesh (bio-materials, size and shape) and fixation methods (titanium tacks, absorbable tacks, fibrin glue) are considered crucial issues to achieve optimal results and reduce complication and recurrence rates [4,5,9–12]. Lightweight meshes are often considered the first choice for hernia repair by many authors, since the evidence that the decreased density of the non-absorbable material could reduce the “foreign-body response,” improve abdominal wall compliance, cause less shrinkage and enhance the integration in host tissues [13–15]. Titanium helicoidal tacks are still considered the “gold standard” for mesh fixation [16–20]. However, several complications caused by these tacks have been reported (adhesion formation and bowel perforation). Furthermore, these devices may be involved in the genesis of some post-operative pain. Recently, absorbable tacks have been introduced [21] in combination with lightweight meshes, under the assumption that permanent fixation is no longer needed after the mesh is integrated within host tissues, potentially avoiding some of the above-described tack-related complications. Nevertheless, the efficacy of the use of absorbable tacks has never been compared to titanium tacks in a prospective analysis: only a few observational studies report similar results during a mid-term post-operative follow-up [21–23].

The Italian Society of Endoscopic Surgery recently carried out a Consensus Conference with a systematic literature search on this topic and concluded that “*mesh fixation with spiral tacks should be considered the standard method of fixation in laparoscopic ventral/incisional hernia repair*” and that “*there is not enough evidence to make any recommendation in favor or against the use of the absorbable fixing devices*” [24]. Also, the International Endohernia Society published its evidence-based guidelines and came to similar conclusions. Both guideline panels found very little literature about the role of absorbable fixing devices and stressed the need for further research about it [25]. In fact, the current evidence on the use, safety and efficacy of absorbable tacks for mesh fixation is still based on few retrospective studies giving inconclusive results [21,22], and a recent report from a National Register [26], which concludes that the use of absorbable tacks is related to an increased risk of hernia recurrence. The latter deals with a very large number of patients but does not provide any information either about the type of mesh or the specific kind of tacks used. The absence of this crucial information limits the usefulness of the study. The direct comparison of absorbable tacks with the current “gold standard,” represented by titanium tacks appears to be the best way to assess their efficacy and safety.

Our study primary objective is to determine if absorbable tacks are non-inferior to titanium tacks for lightweight mesh fixation during LIVHR in the end point of recurrence rate, considering a follow-up of 3 years.

The secondary objectives are to evaluate the possible superiority of the absorbable tacks in improving post-operative stay, complications, pain and comfort.

Our choice of a non-inferiority trial design was based on the assumption that some of the complications related to the use of metal spiral tack could be avoided by the use of absorbable devices if their non-inferiority to the reference treatment (metal tacks) is demonstrated.

This protocol has been prepared in accordance with Standard Protocol Items: Recommendations for Interventional Trials (SPIRIT) [27] and the Consolidated Standards of Reporting Trials (CONSORT 2010) [28], taking into accounts its Extension for Nonpharmacologic Treatment Interventions [29] and the CONSORT Statement for Reporting Noninferiority and Equivalence Trials [30].

## Methods/Design

### Trial design, locations and organizational structure

The TACS trial is a multicentric prospective randomized study. It will involve five surgical units having extensive experience in both metallic and absorbable tacks for LIVHR (this means that each surgeon participating in the study usually performs more than fifty procedures per year with the devices used in the study).

The Reference Center (General Surgery Unit, ICOT Hospital, Latina, Italy) will coordinate the study and handle the data analysis and interim evaluations.

The study will develop according to the *Helsinki Declaration*. It obtained the Ethical Committee approval (Independent Ethical Committee “Lazio 2”) on 26 May 2014 prior to registration (reference number asl_lt/15675/A001/2014)*.*

### Study population and eligibility criteria

All consecutive patients referred to the involved surgical units will be checked to determine eligibility, by one dedicated team member, according to the inclusion and exclusion criteria specified in an additional file (see Additional file 1).

All the eligible patients will be consecutively enrolled in the study until the sample size of 220 patients is reached. The included patients will be divided into two groups:

- Group A: patients submitted to LIVHR by lightweight polypropylene mesh fixed by titanium helicoidal tacks (control group);
- Group B: patients submitted to LIVHR by lightweight polypropylene mesh fixed by absorbable tacks (study group).

Each patient will be introduced to the trial by a member of the research group and receive an explanation of the study protocol (including random assignment of the fixation device that will be used during surgery). A specific informed consent regarding participation in the trial, randomization and explanation of the laparoscopic hernia repair, with no disclosure about the fixation device will be obtained and signed before enrolling in the study.

### Randomization, allocation concealment, and blinding

A simple 1:1 allocation ratio randomization (without any restriction) will be guaranteed by an on-line computerized random generator. Patients will be randomly assigned to either Group A or Group B in the operating room, just before surgery.

Patients will be kept blind to the allocation status. Furthermore, post-operative controls will be performed at each center by a physician not directly involved in the study. He will be kept blind to the particular device used on each patient. Due to the nature of the intervention, the operating staff cannot be blinded to allocation but will be instructed not to disclose the allocation status of the participant at any time.

### Intervention, preoperative and post-operative management

All patients will be assessed preoperatively by clinical examination and abdominal computed tomography (CT) or ultrasound (US) scan to evaluate the size and number of the defect(s).

The devices used in the study (meshes and tacks) will be:

- Physiomesh™ (Ethicon Endo-Surgery, Johnson & Johnson, Inc.), a lightweight polypropylene mesh with double face absorbable layer engineered to be placed on the peritoneal surface
- Protack™ (Covidien Surgical, Mansfield, MA, USA), permanent titanium helicoidal tacks
- Securestrap™ (Ethicon Endo-Surgery, Johnson & Johnson, Inc.), “U”-shaped absorbable tacks made by polydioxanone, completely resorbed by hydrolysis within 12–18 months after implantation

All the procedures will be carried out using the same surgical technique, already described in previous studies [22,23]. In particular, there will be careful adhesiolysis of the entire abdominal wall, a search for additional defects, overlapping the defect for at least 3 cm on each side, setting the tacks at the distance of 1.5 cm in a double crown configuration [31] and no use of transfixed sutures or glue. After surgery, compressive dressing will be placed for 4 weeks.

Each surgeon will be provided with a precise description of the standardized technique and a checklist of the aforementioned necessary technical items to be controlled, filled for every patient and turned back to the Reference Center. Adherence to the protocol will be assessed by review of the procedure videos: all centers will share the videos with the Reference Center. The operations performed by the Reference Center will be examined by the primary surgeon of another participating center who will check that all items on the checklist have been correctly performed.

A member of the operating team will note the patients and intervention characteristics in an online database including the following parameters:

- Patient characteristics (sex, age, comorbidities, personal therapy)
- Hernia features (location and size)
- Mesh size
- Type of tacks (absorbable/non-absorbable)
- Number of tacks used
- Operating time
- Post-operative hospital stay

Post-operative complications during the index admission (according to the Clavien-Dindo classification) [32].

Acute post-operative pain (using the Visual Numeric Scale (VNS) for pain classification) [33].

Pain management (use of analgesic drugs).

There will be no difference in preoperative or post-operative care between the two groups. All patients will receive 1 dose (1 g) of paracetamol given intravenously at surgery and 2 more, each at 8-hour interval. Additional need for pain management will be noted on the records. The follow-up will be scheduled as follows:

- 1 week (clinical evaluation)
- 4 weeks (clinical evaluation)
- 6 months (clinical evaluation or phone interview)
- 12 months (phone call and eventual clinical evaluation)
- 24 months after surgery (phone call and eventual clinical evaluation)
- 36 months after surgery (phone call and eventual clinical evaluation)

During the follow-up, hernia recurrence (with eventual imaging studies) and post-operative complications will be checked. The following data will be also evaluated, using the licensed Carolinas Comfort Scale (CCS) [34,35]:

- Pain (during resting, during daily activities, during walking, during exercise):
- Pain control by use of analgesic drugs (which kind of drug, which dosage)
- Pain at physical examination

### Outcome parameters

The primary end point of the study is:

- comparison of the results of the use of metallic and absorbable tacks to fix lightweight polypropylene meshes during LIVHR, in terms of hernia recurrence, to test the hypothesis of non-inferiority of absorbable tacks when compared to titanium tacks.

The endpoint will be measured by the recurrence rate at 3 years; interim evaluations will be done at 1-year and 2-year follow-up.

The secondary end point is:

- comparison of intraoperative and early post-operative outcomes, in terms of surgical complications, post-operative stay, comfort and pain

The comfort and pain parameters will be measured using the licensed CC S; surgical complications will be evaluated by the Clavien-Dindo classification [32] and on the basis of reoperation and readmission rates (1–2 and 3 years).

### Statistical methods

The sample size calculation is based on the primary end point. A sample size of 110 in each group permits the achievement of a study power of 80 %, to detect a non-inferiority margin difference between the devices used. The reference group proportion is 0.07. The treatment group proportion is assumed to be 0.13 under the null hypothesis of inferiority. The statistic test used will be the one-sided Z test (unpooled). The significance level of the test will be targeted at 0.05.

The same sample size will also be adequate to evaluate the secondary endpoints.

Statistical analysis will be performed at 1, 2 and 3 years after the first patient’s enrollment by a statistician affiliated with the Reference Center.

### Dissemination policy

The results of the TACS trial will be presented at international medical meetings concerning the corresponding fields of interest. Publications are planned in surgical scientific journals. The results will be disseminated regardless of the magnitude or direction of the measured effects.

### Good clinical practice and ethical approval

The study will develop according to the *Helsinki Declaration*. It obtained the Ethical Committee approval (Independent Ethical Committee “Lazio 2”) on 26 May 2014 prior to registration (reference number asl_lt/15675/A001/2014). Any modifications to the protocol that may impact on the conduct of the study, potential benefit to the patient or may affect patient safety will require a formal amendment to the protocol and a new approval of the Ethical Committee according to local regulations.

## Discussion

In recent years, lightweight meshes have been proposed for the treatment of abdominal wall hernias, both in open and laparoscopic repair, to reduce the mesh-related and/or mesh fixation-related complications [13]. Decreasing the density of non-absorbable material, a reduced “foreign-body response” has been demonstrated. This results in an improved abdominal wall compliance and less mess shrinkage, thus allowing a better integration of the mesh in the host tissues [14,15].

This “lightweight approach” is also based on the results of studies concerning the abdominal wall biomechanics [36–39].

Among the large number of meshes and materials utilized for LIVHR, macropore lightweight polypropylene has been demonstrated to be more efficient than other materials in terms of inflammatory response and mesh integration in host tissues [38–42].

A crucial issue in LIVHR is the choice of the mesh fixation method. Titanium helicoidal tacks are most frequently used, in a double crown configuration [43–46]; they guarantee excellent fixation strength [46] but remain in the body permanently, and have been associated with severe complications. Dense adhesion formation, erosion of tacks in hollow viscera and the formation of so-called "tack hernias” have been reported [16,17,47]. However, the most clinically relevant negative aspect appears to be the increased acute and chronic post-operative pain, as reported by several studies [22,23,40–42]. Furthermore, permanent fixation devices seem not to be necessary while using lightweight macropore meshes, as they become integrated into the abdominal wall and may not need to be permanently secured [22,23]. Nevertheless, titanium tacks remain the most common fixation device for LIVHR [47,48]. Recently, absorbable tacks have been introduced [21] to improve biocompatibility, reduce the risk of endoperitoneal adhesions due to the presence of metallic tacks, and reduce mid-term and long-term post-operative pain. The rationale for the use of absorbable tacks in LIVHR for lightweight macropore mesh fixation is mainly the biomechanical mesh features since the integration of these prostheses into the abdominal wall may not need permanent fixation devices. To date, only 2 studies dealing with the clinical use of absorbable tacks with lightweight mesh have been published [21,22]. They report similar intraoperative and early post-operative outcomes when comparing absorbable and non-absorbable tacks [22]. However, these are observational studies on small series of patients, and the reported results still need to be validated.

The aim of the present trial is to compare the results of the use of metallic and absorbable tacks to fix lightweight polypropylene meshes during LIVHR, in terms of hernia recurrence, in order to test the hypothesis the non-inferiority of absorbable tacks when compared to titanium tacks.

Furthermore, the authors will compare acute and chronic post-operative pain after the use of titanium and absorbable tacks.

If the non-inferiority of the absorbable fixation devices was assessed, their use could be encouraged and their possible advantages in terms of reduction of complications and post-operative pain could be further investigated.

### Trial registration and protocol version

Primary registry and trial identifying number*: ClinicalTrials.gov*NCT02076984.

Date of registration in the primary registry: 5 June 2014.

Protocol version: version 1.1 issued on 20 January 2015.

### Trial status

Patient enrollment started on 1 June 2014 after tEthical Committee approval.

## Additional file

Additional file 1:
**Table showing inclusion and exclusion criteria for eligibility in the study.**

## Abbreviations

CCS: Carolinas Comfort Scale
CT: computed tomography
LIVHR: laparoscopic incisional/ventral hernia repair
US: ultrasound
VNS: Visual Numeric Scale
