# Supplementary material for: Titanium versus absorbable tacks comparative study (TACS): a multicenter, non-inferiority prospective evaluation during laparoscopic repair of ventral and incisional hernia: study protocol for randomized controlled trial
Source: Trials. 2015 Jun 4;16:249. doi: 10.1186/s13063-015-0779-x (PMC4460685; doi:10.1186/s13063-015-0779-x)
Supplement: Additional file 1: — Table showing inclusion and exclusion criteria for eligibility in the study. [file 13063_2015_779_MOESM1_ESM.doc]

|  | **INCLUSION CRITERIA** | **EXCLUSION CRITERIA** |
| --- | --- | --- |
| ***HERNIA SITE**** | M2-M3-M4-L2-L3 | M1-M5-L1 |
| ***HERNIA WIDTH**** | W1-W2 | W3 or more |
| ***HERNIA FEATURES*** | Primary hernia; incisional hernia | Recurrent hernia |
|  |  | Incarcerated or strangulated hernia |
| ***INTRAOPERATIVE FINDINGS*** |  | Contaminated field during surgery |
|  |  | Contemporary bowel resection |
|  | BMI < 35 | BMI > 35 |
| ***AGE*** | Between 18 and 75 | < 18; >75 |
| ***COMORBIDITIES*** |  | Severe COPD |
|  |  | Uncontrolled diabetes (glycated Hb >7) |
|  |  | Child B or C cirrhosis |

**Additional File 1: Inclusion and exclusion criteria**

*: According to: Muysoms FE, et al. Classification of primary and incisional abdominal wall hernias: Hernia 2009; 13: 407-414
